# Atrioventricular nodal reentry tachycardia treatment using CARTO 3 V7 activation mapping: a new era of slow pathway radiofrequency ablation is under coming

**DOI:** 10.3389/fcvm.2023.1144988

**Published:** 2023-09-29

**Authors:** Enrico Chieffo, Sabato D’Amore, Valentina De Regibus, Cinzia Dossena, Laura Frigerio, Erika Taravelli, Carolina Ferrazzano, Pasquale De Iuliis, Michele Cacucci, Maurizio E. Landolina

**Affiliations:** ^1^ Department of Cardiology, ASST Ospedale Maggiore, Crema, Italy; ^2^ Department of Cardiology, S. Croce e Carle Hospital, Cuneo, Italy; ^3^ Biosense Webster, Johnson & Johnson Medical S.p.A., Pomezia, Italy

**Keywords:** slow pathway, atrioventricular nodal reentry tachycardia, RF ablation, CARTO 3 version 7, activation map, Confidense module

## Abstract

**Background:**

Slow pathway (SP) ablation is the cornerstone for atrioventricular nodal reentry tachycardia (AVNRT) treatment, and a low-voltage bridge offers a good target during mapping using low x-ray exposure. We aimed to assess a new tool to identify SP by activation mapping using the last CARTO3® version, i.e., CARTO PRIME® V7 (Biosense Webster, Diamond Bar, CA, USA)

**Methods and results:**

Right atrial septum and triangle of Koch 3D-activation map were obtained from intracardiac contact mapping during low x-ray CARTO 3® procedure. In 60 patients (mean age 60.3 ± 14.7, 61% females) undergoing ablation for AVNRT, an automatic activation map using a DECANAV® mapping catheter and CARTO® Confidense™, Coherent, and FAM DX software modules were obtained. The SP was identified in all patients as the latest atrioventricular node activation area; RF catheter ablation (RFCA) in that region elicited junctional beats. The mean procedural time was 150.3 ± 48.3 min, the mean fluoroscopy time exposure was 2.9 ± 2 min, the mean dose-area product (DAP) was 16.5 ± 2.7 cGy/cm^2^. The mean number of RF applications was 3.9 ± 2, the mean ablation index was 428.6 ± 96.6, and the mean contact force was 8 ± 2.8 g. There were no adverse event during the procedure, and no AVNRT recurrences occurred during a mean follow-up of 14.3 ± 8.3 months.

**Conclusion:**

Ablation of the SP by automatic mapping using Confidense™, Coherent, and FAM DX software modules is an innovative, safe, and effective approach to AVNRT ablation. The CARTO3® V7 system shows on a 3D map the latest AV node activation area during sinus rhythm allowing low fluoroscopy time and highly effective RFCA.

## Introduction

Atrioventricular nodal reentry tachycardia (AVNRT) is the most common supraventricular tachycardia in clinical practice (1), and catheter ablation is the treatment of choice for symptomatic patients (2) improving quality of life. The success rate is high (91%–99% of patients) and recurrences are seen in 5%–9%, considering both radiofrequency (RF) and cryogenic application, and AV block is a rare complication (about 1% of the patients) (3, 4). The slow pathway (SP), generally located in the lower one-third of the Koch triangle in the postero-septal region anterior to the ostium of the coronary sinus (CS), is targeted during ablation using either RF energy or cryoablation, with the aim of rendering the tachycardia not inducible at the end of procedure. During effective ablation, RF energy elicits junctional beats whereas cryoenergy results in non-inducibility with or without echo beats during freezing. Recently, non-fluoroscopic 3D mapping has been used to identify in the postero-septal region a low-voltage bridge as a new target for SP ablation, especially in children (5). The objective of our study was to assess, in a consecutive adult population undergoing SP ablation, how to identify SP location by activation mapping using the last CARTO 3 version, i.e., CARTO PRIME V7 (Biosense Webster, Diamond Bar, CA, USA) (6). The study also aimed to evaluate whether this technique may have an impact on the complexity of the procedure, on the success rate, and on the risk of complications.

## Methods

We performed a single-center prospective observational study in 60 consecutive patients (females 61%, mean age 60.3 ± 14.7 years) who underwent catheter ablation for AVNRT between May 2020 and November 2021. All patients had structural normal heart confirmed by echocardiogram. The study was in accordance with the guiding principles of the Declaration of Helsinki; written informed consent was obtained prior to the ablation procedure. Each patient provided informed consent for data collection and analysis.

### Electrophysiological study

In all patients, a low x-ray exposure protocol was applied during the Electrophysiological study (EP). The procedure was performed in conscious sedation without general anesthesia. Antiarrhythmic drugs were discontinued for at least five half-lives before the procedure. A quadripolar 2-5-2 mm and a decapolar 2-8-2 mm (DECANAV®, Biosense Webster, CA, USA) catheters were inserted into the left femoral vein and positioned at the His bundle area and in the coronary sinus, respectively, after fast anatomical mapping (FAM) reconstruction. A SMARTTOUCH 4 mm ablation catheter (Biosense Webster©, Inc., Irvine, CA, USA) was inserted into right femoral vein and positioned in the right ventricle (RV) without x-ray exposure. Incremental atrial pacing and programmed atrial or ventricular stimulation were delivered to induce supraventricular tachycardia: the same stimulation protocol was repeated under isoproterenol infusion (0.01–0.04 μg/kg/min) in case of non-induction in baseline condition. An atrial-His jump was considered diagnostic of dual AV node physiology if a sudden prolongation of AH interval by 50 ms was documented following a shortening of the coupling interval of the atrial extrastimulus by 10 ms. During tachycardia, RV reset and entrainment maneuvers were delivered to exclude accessory pathway/atrial tachycardia and to confirm AVNRT. During sinus rhythm para-Hisian pacing was performed to exclude concealed antero-septal accessory pathway. Typical (slow–fast) AVNRT was defined by an atrial-His/His-atrial ratio (AH/HA) >1 and HA interval ≤70 ms; atypical AVNRT (slow–slow and fast–slow) was defined by a delayed retrograde atrial activation HA > 70 ms (2, 7). In the absence of inducible tachycardia, the evidence of an AH jump with an echo beat together with previous electrocardiographic records of a narrow complex tachycardia consistent with AVNRT were considered reasonable features suggestive of this tachycardia.

### CARTO 3 mapping

**Figure 1:**
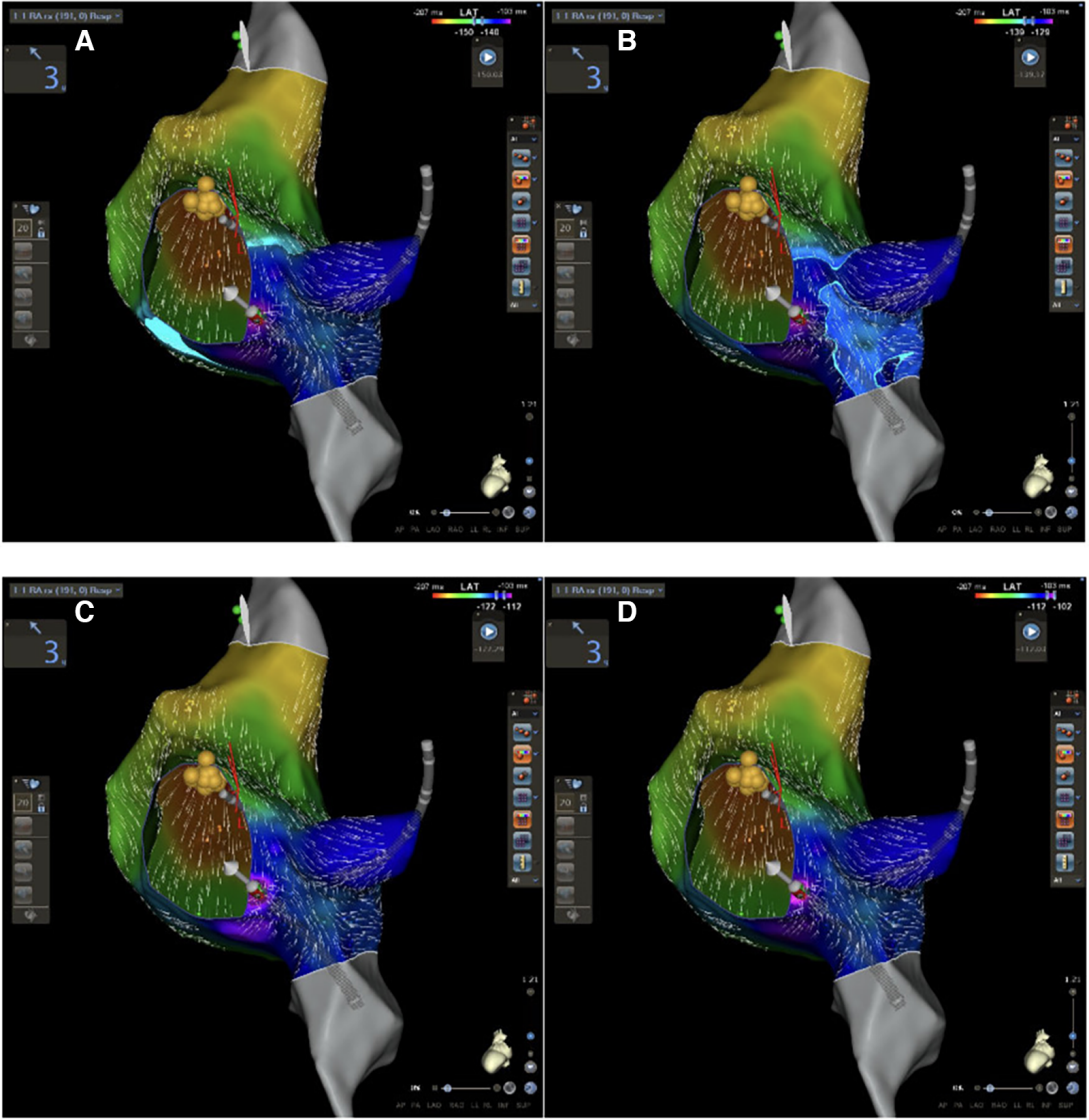
CARTO 3 PRIME V7 atrial and Koch's triangle activation during sinus rhythm in LAO view. (**A**–**D**) Progressive activation of Koch's triangle: slow pathway is located at the area of latest activation (purple area in **D**).

Using the DECANAV catheter and the FAM DX© module (Biosense Webster©, Inc., Irvine, CA, USA), FAM of the right atrium, coronary sinus, and Koch triangle were performed and a voltage gradient and late activation time (LAT) maps were created during sinus rhythm generating a 3D color endocardial gradient map. After FAM reconstruction, quadripolar and decapolar catheters were positioned, respectively, on the His bundle and in the coronary sinus without fluoroscopy exposure. Using Confidense and Coherent© modules, a window of interest (WOI) from −300 to −80 ms before the P wave onset was set. Tissue proximity, LAT stability (5), and cycle length range (925–1,100 ms) were activated too. His potential was accurately marked on the map but excluded during LAT acquisition. The latest atrial activation in correspondence with the Koch triangle at the LAT map was considered a marker of the SP position and was used as a target for ablation (Figure 1). A low-voltage bridge was defined from the voltage gradient map as previously described by Drago et al. (5, 8).

### Ablation procedure

**Figure 2:**
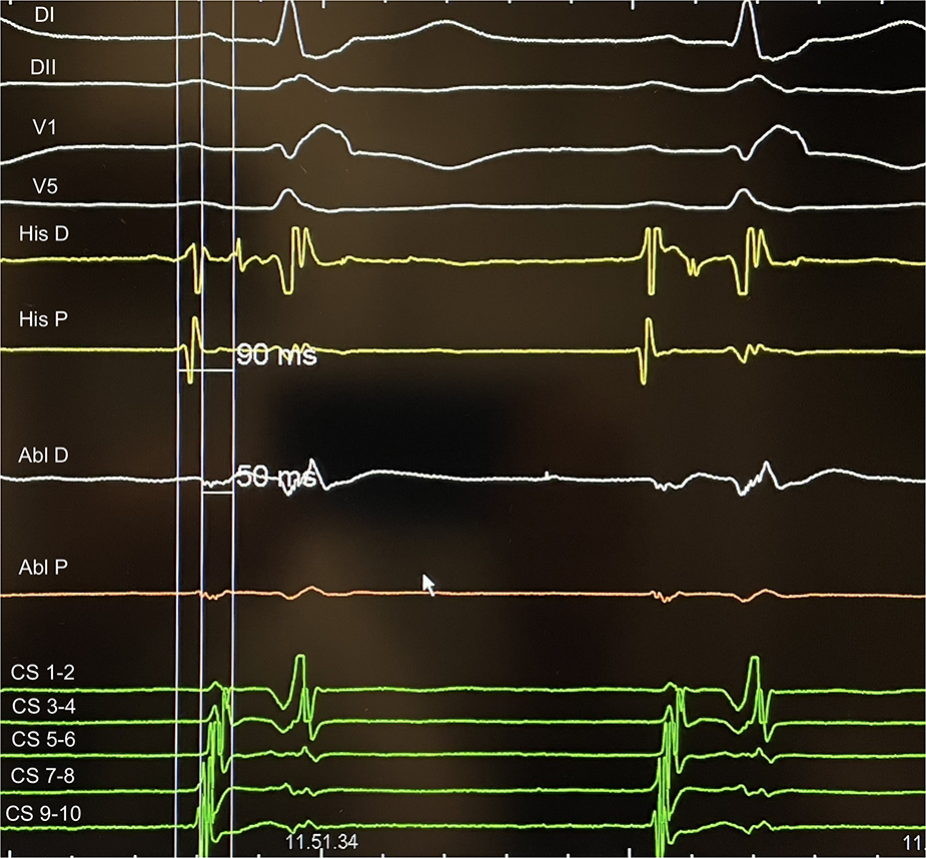
An example of AH measurement on His catheter and at the ablation site during slow pathway ablation: the difference represents delay of the local atriogram to His atriogram (40 ms in this case).

Once the diagnosis of AVNRT was established, the ablation catheter was moved from RV to Koch Triangle. In correspondence of Koch triangle's latest activation area identified with the techniques described above (Figure 1), a “hump and spike” signal (9, 10) was recorded during sinus rhythm, and the maximum local atrial-His delay (ms) and the local distance (mm) from the His bundle were evaluated (Figure 2). RF energy [power 30 W, not irrigated tip, temperature setting of 55°C, minimum contact force (CF) 5 g, up to 60 s] was delivered in correspondence to the latest activation area to obtain junctional beats with 1:1 retrograde ventriculo-atrial (VA) conduction. In case of absence of VA retrograde conduction during junctional rhythm, RF delivery was immediately interrupted. Once junctional rhythm with VA conduction was recorded, energy delivery was continued up to 60 s or until junctional rhythm cessation. Following ablation, arrhythmia induction at baseline and under isoproterenol infusion was attempted. End points for ablation success were non-inducibility of AVNRT and elimination of atrial-His jump; in case of modulation of slow pathway, single atrial-His jump eventually followed by single reentry was tolerated without tachycardia induction (6).

### Follow-up

After successful ablation, patients were discharged within 24 h on aspirin and no antiarrhythmic drugs. All patients were followed up for at least 12 months; follow-up visit and 24 h ECG Holter were programmed at 3 and 12 months. No patients were lost to follow-up. Documentation of AVNRT at 12 lead ECG or 24 h ECG Holter or emergency room admission for symptoms compatible with supraventricular tachycardia were considered arrhythmia recurrences.

### Statistical analysis

Descriptive statistics (mean ± SD, counts and percentages) were used to summarize the data. A two-sample Student's *t*-test were used to compare distance (mm) from ablation point to His potential between subgroups of patient (<75 and ≥75 years). Receiver operating characteristic (ROC) curve analysis was performed to identify the optimal cut-off of the ablation index (AI) and CF for predicting effective radiofrequency ablation.

## Results

The results are reported in Table 1. All patient enrolled in our study underwent electrophysiological study and ablation procedure. No major complications occurred during and after the procedure. Procedural success rate was 100%, and there were no tachycardia recurrences during a mean follow of 14.3 ± 8.3 months. No acute or late AV block was documented. Slow–fast AVNRT was induced in 58 (96%) patient, slow–slow AVNRT was documented in 1 (2%) patient, and a fast–slow tachycardia was found in 1 (2%). A residual SP function with an echo beat was remained at 40% of clinical cases (24 patients) after RF application: no AVNRT recurrences were reported at clinical follow-up. The mean procedural time was 150.3 ± 48.3 min, the mean fluoroscopy time exposure was 2.9 ± 2 min, and the mean dose-area product (DAP) 16.5 ± 2.7 cGy/cm^2^. The mean number of RF applications was 3.9 ± 2, the mean ablation index was 428.6 ± 96.6, and the mean contact force was 8 ± 2.8 g.

**Table 1:** Demographic and procedural characteristics of the overall population.

Overall population	
Age (years)	60.3 ± 14.7
Sex (M/F, %)	39/61
Symptomatic (%)	100
Access to ER (%)	46.6
Tachycardia CL (ms)	375 ± 65.1
AVNRT	
Slow–fast (%)	96
Fast–slow (%)	2
Slow–slow (%)	2
Slow pathway	
Ablation (%)	60
Modulation (%)	40
Cardiomyopathy (%)	1.5
Fluoroscopy time (min)	2.9 ± 2
Radiofrequency total time (s)	200 ± 121
Procedural time (min)	150.3 ± 48.3
DAP (cGy/cm^2^)	16.5 ± 2.7
RF applications	3.9 ± 2
Follow-up (months)	14.3 ± 8.3
Acute procedural success (%)	100
Arrhythmic recurrences at follow-up (%)	
3 months	0
12 months	0
AV block (%)	
Acute	0
Late	0
AH–His cath (ms)	96.3 ± 27
AH (abl site) (ms)	52.3 ± 20
AI (ablation site)	428.6 ± 96.6
CF (g)	8 ± 2.8
Distance Abl site–His bundle (mm)	18.6 ± 4.5

DAP, dose-area product; AI, ablation index; CF, contact force.

**Figure 3:**
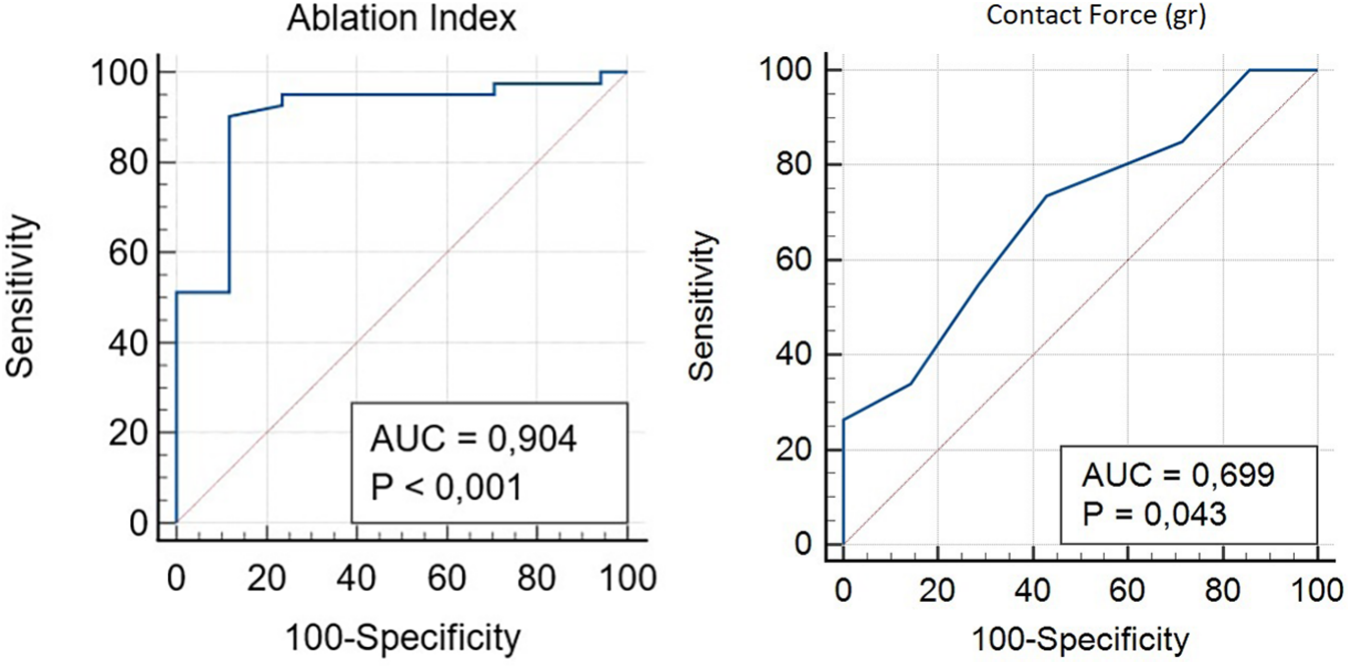
ROC curve analysis was performed to identify the optimal cut-off of the AI and CF for predicting effective radiofrequency ablation.

Distance from the ablation site to the His bundle was 18.6 ± 4.5 mm, and no significant differences were found between young and old patients (*p* = 0.64), after dividing our population into two groups and considering 75 years elderly threshold. Delay of the local atriogram to His atriogram was 44 ± 7 ms (Table 1). Ablation points were located at the postero-septal area (88%), at medio-septal area (6%), and at coronary sinus ostium (6%). No ablation site was located inside the coronary sinus, at the antero-septal region or at the postero-septal aspect of the mitral valve. We found that A/V ratio at the target point was 1/2 in all our procedure (Figure 2). Furthermore, ROC curves were created to identify the cut-off value for AI (>380, AUC: 0.90, *p* < 0,001, sensitivity 90.2, specificity 88.2) and for CF (>6 g, AUC: 0.69, *p* = 0.04, sensitivity 73.5, specificity 57.1) (Figure 3).

## Discussion

In 60 consecutive patients, undergoing EP study and catheter ablation for AVNRT, was used a new mapping tool by Biosense CARTO 3® PRIME® V7 system including Confidense, Coherent, and FAM DX modules. The reduction of radiological exposure in the electrophysiology lab has become a decisive point for everyday activity due to the stochastic and deterministic effects that x-rays could have on our patient's health (11). For this reason, our ablation technique was different from the conventional one: we used the latest technological innovations by Biosense CARTO 3 to reduce radiological exposure and cancer risk without reducing the effectiveness of ablation (absence of recurrences) or increasing procedure-related complications. As described by Mascia and Giaccardi (12), electrophysiologists should ensure that x-ray exposure is low as reasonably achievable without sacrificing quality of care: zero and near-zero fluoroscopic approaches represent a milestone for cancer prevention in ablation procedure. For these reasons, we used the Confidense, Coherent, and FAM DX modules that allowed a high-definition mapping as well as an accurate selection of electrical activation points under low radiological exposure. In particular, using DECANAV® catheters and CARTO® 3 FAM DX software, we performed a fast electroanatomical reconstruction and activation map of the right atrium/Koch's triangle/tricuspid valve in sinus rhythm by creating a high-density mapping points without using an ablator catheter. The CARTO® 3 Confidence Module allowed us to collect a large number of atriograms by selecting them and discarding points not pertinent to the ongoing reconstruction. CARTO® 3 Coherent Module integrated vector and velocity information to every electroanatomical point in a vector map allowing us to identify easily the ablation site.

These new CARTO® 3 system technologies may offer a fast method to evaluate Koch's triangle activation and to perform AVNRT ablation. All atriograms were annotated excluding the His bundle signal to create an annotation map of the right atrium, in particular, of Koch's triangle: in this way, it was possible to identify the latest activation of Koch's triangle which may be an expression of the AV nodal slow pathway. This hypothesis agrees with the first descriptions of Spach and Josephson (13, 14) according to which AV nodal slow pathway cannot be identified by a single endocavitary signal (such as His bundle), and it is not a defined region with a set of specialized cells but a wide variation of a local non-uniform anisotropic conduction. No areas of conduction block were identified as previously described by Pandozi et al. (15). A convergence of the activation front has been identified in correspondence with the slow conduction pathway: a wavefront coming from the apex of Koch's triangle (fast conduction pathway) and another coming from the base (tricuspid valve), which is activated laterally along the cavo-tricuspid isthmus (Figure 1).

Our mapping and ablation approach using new technologies for identifying the area of slow conduction seems to militate in this direction (Figure 1). We suggest that this new method has several advantages: it is simple (it uses the same number of catheters as a traditional ablation), fast (it takes a few minutes more than traditional methods that are compensated by shorter ablation time), reproducible (in all our cases it was possible to identify it the latest activation of Koch's triangle), and safe (no temporary or permanent AV blocks or other complications occurred). Furthermore, our study suggests new cut-off settings for AVNRT ablation procedure: a successful procedure needs an ablation index of more than 380 and a CF of more than 6 g (Figure 3). No AI or CF values were previously described for AVNRT ablation procedures. Our approach with low x-ray exposure achieved an average DAP of 16.5 ± 2.7 cGy/cm^2^, it corresponds to an effective dose (ED) of 0.032 ± 0.005 mSv for an adult patient calculated with the formula DAP (Gy) × 0.2. When compared with the ED data reported in the work of Picano and Piccaluga (11) for an AVNRT ablation (ED 4.4 mSv), it represents a big step forward in terms of x-ray exposure reduction for electrophysiologists and patients, equal to an additional lifetime risk of fatal and non-fatal cancer between 1/10^5^ and 1/10^6^.

The follow-up period is not excessively long due to the recent introduction of these CARTO 3 system tools, and we have not documented acute or late AV conduction blocks (16, 17): it was never necessary to perform the ablation in proximity of areas where a far field of the Hisian potential was recorded or to redo the ablation procedure for arrhythmic recurrences.

## Conclusions

Activation mapping of Koch's triangle with new CARTO PRIME® V7 modules (Confidense, Coherent and FAM DX) during a procedure of AVNRT ablation allows to correctly identify the slow conduction pathway, to perform an effective ablation and to minimize the risk of complications such as atrioventricular block even in cases without a low-voltage bridge identification. Furthermore we suggest new cut-off settings for AVNRT ablation procedure such as the effective ablation index, the delay in ms of target signal compared to the Hisian atriogram and the mean catheter force at the ablation site. As expected, this new method significantly reduces radiological exposure which is a current theme in electrophysiological labs.

## Study limitation

The limitations of the current analysis are the same as those of other single-center observational studies, such as potential bias in patient selection and lack of a control group. Nevertheless, possible bias is mitigated by the fact that patients were consecutively and prospectively included. It may not be ruled out that the absence of recurrences could be either due to the relatively limited number of patients or the duration of follow-up. Moreover, the absence of recurrence could have influenced and limited the relevance of some mapping and activation parameters such as the presence of areas of slow conduction inside the coronary sinus or at postero-septal aspect of the mitral valve. Thus, larger multicenter studies could be helpful to evaluate better the relevance of this new mapping method for AVNRT ablation.

## Data Availability

The raw data supporting the conclusions of this article will be made available by the authors, without undue reservation.
